# *Tetrastigma hemsleyanum* Diels et Gilg Flavonoids Against Acute Lung Injury Via Block NLRP3 Inflammation

**DOI:** 10.1155/mi/2279402

**Published:** 2026-03-08

**Authors:** Lianghui Zhan, Lingling Li, Xiaojun Wu, Xuechun Jiang, Jie Zhou, Sheng Zhu, Changcheng Shu, Jinbao Pu, Weiqing Liang

**Affiliations:** ^1^ Tongde Hospital of Zhejiang Province, Hangzhou, Zhejiang, 310014, China, zjtongde.com; ^2^ Center for Medicinal Resources Research, Zhejiang Academy of Traditional Chinese Medicine, Hangzhou, Zhejiang, 310014, China, zcmu.edu.cn; ^3^ Zhejiang Key Discipline in Traditional Chinese Medicine for Pharmaceutical Botony, Hangzhou, Zhejiang, 310014, China; ^4^ Zhejiang Engineering Research Center for Quality Assessment and Development of Dao-di Herbs, Hangzhou, Zhejiang, 310014, China; ^5^ College of Pharmacy, Zhejiang Chinese Medical University, Hangzhou, Zhejiang, 310014, China, zcmu.edu.cn; ^6^ Zhejiang Guangsheng New Energy Co., Ltd., Quzhou, Zhejiang, 324000, China; ^7^ State Key Laboratory for Quality Ensurance and Sustainable Use of Dao-di Herbs, Beijing, 100700, China

**Keywords:** acute lung injury, molecular docking, network pharmacology, NLRP3 inflammasome, Sanyeqing

## Abstract

**Background:**

*Tetrastigma hemsleyanum* Diels et Gilg (Sanyeqing [SYQ]), a traditional anti‐inflammatory herb, has been used to treat respiratory disorders.

**Aims:**

To elucidate the mechanism of SYQ flavonoids in mitigating acute lung injury (ALI).

**Materials and methods:**

An integrated approach combined network pharmacology, HPLC, lipopolysaccharide (LPS)‐induced ALI mouse models, and NOD‐like receptor thermal protein domain associated protein 3 (NLRP3)‐activated cellular assays (LPS + nigericin). NLRP3 knockdown (siRNA) and molecular docking were employed for mechanistic validation.

**Results:**

Network pharmacology and HPLC identified procyanidin B1 and catechin as core active compounds targeting the NLRP3 inflammasome pathway. Animal experiments demonstrated that SYQ flavonoids can significantly alleviate pathological damage to lung tissue, reduce pulmonary edema, and inhibit the expression of pro‐inflammatory factors, thereby exerting a protective effect against ALI. Furthermore, SYQ flavonoids exhibited protective effects against ALI by downregulating the expressions of interleukin (IL)‐1β, IL‐18, NLRP3, ASC, and caspase‐1. Notably, when NLRP3 was knocked down using siRNA technology, it had no significant effect on the levels of IL‐1β and IL‐18, indicating that their therapeutic effects are mediated through the NLRP3 pathway. Finally, molecular docking confirmed that both catechin and procyanidin B1 exhibit strong binding affinities with NLRP3, providing a molecular basis for their targeted inhibition of the NLRP3 inflammasome.

**Conclusion:**

SYQ flavonoids alleviate ALI by specifically inhibiting the NLRP3 inflammasome, providing a mechanistic basis for its traditional use in lung inflammation.

## 1. Introduction

Acute lung injury (ALI) arises from dysregulated systemic inflammation triggered by direct/indirect pulmonary insults, often exacerbated by respiratory viruses or bacterial pathogens [1, 2]. This condition features acute diffuse inflammatory damage, causing increased vascular permeability, neutrophil infiltration, pulmonary edema [3], and impaired ventilation. With incidence rates reaching 30%–40% mortality [4], ALI remains a critical clinical challenge despite therapeutic advances, as no effective pharmacologic interventions exist [5]. The urgent need for novel treatments persists.

Growing evidence supports natural products as promising interventions for ALI [6], with flavonoids demonstrating particular potential due to their anti‐inflammatory and lung‐protective properties [7]. *Tetrastigma hemsleyanum* Diels et Gilg (Sanyeqing [SYQ]), traditionally used against inflammatory conditions including ALI [8], contains bioactive flavonoids validated in ALI animal models. However, systematic exploration of SYQ flavonoids’ therapeutic mechanisms, including targets, biological processes (BPs), and metabolic pathways, remains limited.

Bioinformatics has transformed traditional Chinese medicine (TCM) research from a single drug, single‐target paradigm to a comprehensive multicomponent, multitarget, and multipathway approach [9], enabling identification of therapeutic targets and enhancing scientific validation [10].

Therefore, we integrated network pharmacology, HPLC, molecular docking, and experimental validation to elucidate SYQ flavonoids’ mechanism against ALI.

## 2. Materials and Methods

### 2.1. Chemicals and Reagents

SYQ was obtained from Guangsheng Group Co. Ltd (#2‐20120122701, Zhejiang, China). Macroporous resin HPD‐826 (S27514), hematoxylin and eosin (HE, R20570), and Masson staining (R20381) were bought from Yuanye (Shanghai, China). The standards of procyanidin B1 (PS010162), catechin (PS012798), kaempferol‐3‐O‐rutinoside (MUST‐16041507), and astragalin (MUST‐18031820) were purchased from PUSH Bio‐technology (Sichuan, China). Lipopolysaccharide (LPS, L2630) was bought from Sigma Aldrich (Shanghai, China). BEAS‐2B cells (20221121‐02) were bought from Fuheng Biotechnology Co., Ltd (Shanghai, China). Dulbecco’s modified Eagle’s medium (DMEM, PYG0073, BOSTER, Wuhan, China), interleukin (IL)‐1β (Cat. # 1301814V), IL‐6 (Number: Cat. # m1063159V), IL‐10 (Cat. # m1037873V), and tumor necrosis factor (TNF)‐α (Number: Cat. # m1002095V) were bought from Mlbio (Shanghai, China), and lactate dehydrogenase (LDH, A020‐2‐2) was bought from Nanjing Jiancheng Bioengineering Institute (Nanjing, China). JC‐1 (C2006), bicinchoninic acid assay kit (P0010), and MTT (ST316) Lip8000 (C0551) were bought from Beyotime (Shanghai, China). The antibodies of apoptosis‐associated speck‐like protein containing CARD antibody (ASC, DF6304) and IL‐1β (AF5103) were bought from Affinity (Jiangsu, China). The antibodies of IL‐18 (10663‐1‐AP) and NOD‐like receptor thermal protein domain‐associated protein 3 (NLRP3, 68102‐1‐lg) were bought from Proteintech (Wuhan, China). Pro‐caspase‐1 antibody was bought from Abcam (ab179515; Shanghai, China). MonScript RTIII All‐in‐One Mix was bought from Monad (Lot.430530, Wuhan, China). Si‐NLRP3 (1144502530/1144502531) was bought from Sangon Biotech (Shanghai, China).

### 2.2. Preparation of Total Flavonoids From SYQ

A suitable quantity of dried SYQ was weighed accurately and transferred to a conical flask. A 10‐fold volume of 60% (v/v) ethanol was added, and then the mixture was soaked for 30 min. Subsequently, two consecutive extraction cycles were conducted by heating the mixture under reflux in a 90°C water bath for 2 h per cycle. The filtrates were pooled from both extractions and concentrated under reduced pressure to a final concentration of 8 mg/mL. Finally, the concentrated extract was purified using macroporous resin HPD‐826, yielding a total flavonoid content of ~723.6 mg/g.

### 2.3. Analysis of Chemical Components of SYQ Flavonoids

All separations were performed on an Agilent XDB C‐18 column (4.6 mm × 150 mm, 12 nm) at 30°C using the Agilent 1260 Infinity II HPLC‐DAD system. The mobile phase was composed of solvent A (0.085% phosphoric acid aqueous solution) and solvent B (acetonitrile) with a linear gradient: 0–15 min (A: 90%–85%), 20–25 min (B: 85%–80%), 25–45 min (B: 80%–65%), and 45–60 min (B: 65%–0%). The DAD detection wavelength was set at 266 nm. Symmetrical peaks were obtained at a flow rate of 1.0 mL/min with a sample injection volume of 20 μL.

### 2.4. Identification of Differentially Expressed Genes (DEGs) of ALI and Hub Genes

The Gene Expression Omnibus (GEO: www.ncbi.nlm.nih.gov/geo/) datasets were used to search for the mRNA expression profiles of ALI and normal samples (GSE2322 and GSE32707). Differential analysis was performed using the R software package limma (Version 3.40.6) to identify DEGs between the ALI (model group) and normal groups (control group), with thresholds set at |log_FC_| > 1.5 and *p*  < 0.05. The volcano plots and heat maps were generated in R using the ggplot2.

### 2.5. Collection of SYQ Flavonoid Compounds and Targets

The compounds of SYQ flavonoid were collected from these databases, including HERB, CNKI, and PubMed, and then screened for compounds that meet the criteria of OB ≥ 30 and DL ≥ 0.18. The flavonoid compound targets within SYQ were investigated using the Traditional Chinese Medicine Systems Pharmacology Database. Subsequently, the target protein names were transformed into official gene symbol formats with the assistance of UniProt databases.

### 2.6. TCM‐Compounds‐Target‐Diseases and Protein–Protein Interaction (PPI) Network Construction

The interaction between the hub genes of ALI and the targets of flavonoid compounds in SYQ was visualized by Venn diagram. To offer a more comprehensible elucidation of the correlation between three flavonoid compounds in SYQ and ALI, the TCM‐compounds‐target‐diseases network was built, which was performed using Cytoscape software 3.9.1.

The interaction targets were uploaded to the Search Tool for Recurring Instances of Neighboring Genes (STRING) database, with a species restriction to “*Homo sapiens*” and a confidence score set at ≥0.400 in the operation interface. Subsequently, Cytoscape software Version 3.9.1 was utilized for generating a network of potential key targets and conducting a systematic analysis of network parameters.

### 2.7. Gene Ontology (GO) and Kyoto Encyclopedia of Genes and Genomes (KEGGs) Enrichment Analysis

GO and KEGG enrichment analyses were performed to identify hub genes. Significant results (*p* < 0.05 and FDR < 0.25) were visualized using a bubble chart.

### 2.8. Animal Grouping and Treatment

Male C57/BL mice (6–8 weeks, 18 ± 2 g) were sourced from Hangzhou Medical College (License Number SCXK [Zhe] 2019‐0002, SPF). They were housed in cages with ad libitum access to food and water, following a 2‐week quarantine and acclimatization period. All animal procedures were ethically approved by the Animal Ethics Community of Zhejiang Academy of Traditional Chinese Medicine (Ethical Number: 2023‐068), in compliance with European Community Guidelines for Laboratory Animal Care.

All mice were randomly divided into six groups (10 mice in every group): control, model, SYQ flavonoids of high dose (SYQ‐FH, administered 80 mg/kg flavonoids of SYQ), medium dose (SYQ‐FM, administered 40 mg/kg flavonoids of SYQ), low dose (SYQ‐FL, administered 20 mg/kg flavonoids of SYQ), and MCC950 (the inhibitor of NLRP3, 10 mg/kg) groups. SYQ‐F group mice were administered orally for 3 days. MCC950 group mice were injected with MCC950 on the first and third day [11]. LPS (3 mg/kg) was injected into mice half an hour after final administration to induce the ALI model [12]. The mice were sacrificed 3 h after modeling.

### 2.9. Bronchoalveolar Lavage Fluid (BALF) Collection

The abdominal cavity of the mice was opened, and the lungs were exposed. The trachea was intubated, the left lung was ligated, and the right lung was rinsed twice with 1 mL of cold PBS while collecting the BALF. Subsequently, the samples were centrifuged at 4°C and 3500 rpm for 10 min to obtain supernatant.

### 2.10. Lung Wet/Dry (W/D) Weight Measurement

After the mice were sacrificed, lung tissues were removed and immediately weighed to obtain the wet weight. The wet lung tissues were placed in an oven at 60°C for 48 h, after which the dry weight was measured. Finally, the lung (W/D) weight ratio was obtained.

### 2.11. Lung Histopathology

After the mice were sacrificed, the left lung was excised and immersed in 4% formalin for a duration of 48 h. Subsequently, the lung tissue underwent dehydration, embedding, and sectioning (4 μm). Following these procedures, the sections were subjected to HE and Masson staining. Ultimately, microscopic examination was conducted to observe the pathological condition (200×). In addition, the inflammatory pathology of HE‐stained lung tissue was graded and scored according to Supporting Information 1: Table S1.

### 2.12. Inflammation Cytokines

Blood samples were collected from mice via the ocular cavity and lysed at 4°C for 1 h in radio immunoprecipitation assay lysis buffer, followed by centrifugation at 4°C and 3500 rpm for 10 min to obtain supernatant. Inflammatory cytokines, including IL‐1β, IL‐6, IL‐10, and TNF‐α, in serum and BALF were detected by enzyme‐linked immunosorbent assay, and LDH levels were measured according to the manufacturer’s instructions.

### 2.13. Cells Culture and Treatment

BEAS‐2B cells were cultured with DMEM in an incubator at 37°C with 5% CO_2_ for 24 h. Following a 24‐h incubation, the cells were treated with SYQ‐F at different concentrations (10, 5, 2.5, 1.25, 0.625, 0.3125, 0.15625, and 0 μg/mL), or incubated with LPS (1 μg/mL) + nigericin sodium (2.5 μg/mL) or LPS + nigericin sodium + SYQ‐F (10, 5, and 2.5 μg/mL) for 24 h for the following experiments.

### 2.14. MTT Assay

The cells were seeded in a 96‐well plate at a density of 1 × 10^3^ cells/well and incubated for 24 h. After the modeling and drug administration process was completed, each group was replaced with MTT assay (0.5 mg/mL). After 3 h, 150 μL DMSO was added into each well. Finally, the optical density (OD) values were measured at 490 nm.

### 2.15. LDH Assay

Using the LDH assay kit, cells were seeded at a density of 1 × 10^3^ cells/well in 96‐well plates and incubated for 24 h. After treatment, the plates were centrifuged at 1000 rpm for 5 min to collect supernatants. Reaction mixtures (supernatant + kit reagents) were prepared following the protocol and incubated at room temperature for 30 min. Absorbance was then measured at 490 nm to quantify LDH release.

### 2.16. JC‐1

The cells were seeded in a 24‐well plate at a density of 2.5 × 10^5^ cells/well and incubated for 12 h. Once the cells reached ~70%–80% confluency, the culture medium was removed and the cells were treated with serum‐free DMEM, LPS + nigericin sodium, or LPS + nigericin sodium + SYQ‐F (10, 5, and 2.5 μg/mL) for 24 h. The cells were then gently washed with PBS. The JC‐1 stock solution was prepared according to the manufacturer’s instructions and diluted in DMEM medium. Post incubation, the cells were washed with washing buffer to remove excess JC‐1. Fluorescence was analyzed using a fluorescence microscope. JC‐1 emits different colors (green and red) depending on the membrane potential, allowing assessment of mitochondrial health. The results were interpreted based on the fluorescence intensity ratio of red to green, which indicates mitochondrial membrane potential status.

### 2.17. ROS Assay

BEAS‐2B cells were cultured in DMEM supplemented with 10% FBS under standard conditions (37°C, 5% CO_2_). The cells were seeded into 96‐well or 24‐well plates at an appropriate density to achieve ~80% confluency on the day of the experiment. The cells were treated with serum‐free DMEM, LPS + nigericin sodium, or LPS + nigericin sodium + SYQ‐F (10, 5, and 2.5 μg/mL) for 24 h. The working solution was prepared according to the manufacturer’s instructions. Finally, the fluorescence intensity was measured using a microplate reader with excitation/emission wavelengths typically set at 488/525 nm or observed under a fluorescence microscope.

### 2.18. Cell Transfection

BEAS‐2B cells were seeded in a 6‐well plate at a density of 20,000 cells per well. Once the cells reached ~70% confluency, the culture medium was replaced with fresh culture medium. First, serum‐free medium was mixed with si‐NLRP3, and then Lipo8000 transfection reagent was added. The mixture was gently mixed and incubated at room temperature for 20 min. The mixture was evenly distributed into the wells, and the cells were cultured for 3 days. The transfected cells were then used for subsequent experimental procedures.

### 2.19. Western Blot

Lung tissue (80 mg), retrieved from the −80°C refrigerator, and cell samples were both homogenized using radio immunoprecipitation assay lysis buffer. The homogenized samples were incubated at 4°C for 1 h, followed by centrifugation at 12000 rpm to collect the supernatant. Protein concentration was determined using a bicinchoninic acid assay kit. Proteins were separated by sodium dodecyl sulfatepolyacrylamide gel electrophoresis and transferred onto a polyvinylidene fluoride membrane. The membrane was blocked with 5% skim milk for 2 h, and then incubated overnight at 4°C with primary antibodies: ASC (1:1000), IL‐18 (1:2000), NLRP3 (1:1000), pro‐caspase‐1 (1:1000), and IL‐1β (1:500). The following day, the membrane was incubated with secondary antibodies, Goat Anti‐Rabbit IgG(H + L) or Goat Anti‐Mouse IgG(H + L), at a dilution of 1:10000. An image of the membrane was captured using ECL reagents on an imaging system.

### 2.20. Immunofluorescence

The cells were seeded in a 24‐well plate at a density of 2.5 × 105 cells/well and incubated for 12 h. Once they reached ~70%–80% confluency, the culture medium was removed, and the cells were treated with serum‐free DMEM, LPS + nigericin sodium, LPS + nigericin sodium + SYQ‐F (10, 5, and 2.5 μg/mL) for 24 h. The cells were then fixed using paraformaldehyde for about 15–20 min at room temperature. The fixed cells were washed with PBS and then permeabilized by incubating with a permeabilization buffer for about 5–10 min. The cells were incubated with a 5% BSA for 30–60 min to minimize nonspecific binding of antibodies. The ASC and cleaved‐caspase‐1 antibody diluted in blocking solution were added and incubated overnight at 4°C. The cells were washed several times with PBS to remove unbound primary antibodies. A fluorescently labeled secondary antibody was applied for 1 h at room temperature. The cells were washed again with PBS, and then the coverslips were mounted onto a microscope slide using mounting medium. The cells were observed under a fluorescence microscope, and images of the stained cells were captured for analysis.

### 2.21. Quantitative Real‐Time Polymerase Chain Reaction (RT‐PCR)

Total RNA extraction from lung tissue/cells was carried out using Trizol reagent. Subsequently, cDNA synthesis was performed utilizing MonScript RTIII All‐in‐One Mix along with dsDNase. Following this, RT‐qPCR amplification was conducted using MonAmp SYBR Green qPCR Mix to identify NLRP3, IL‐18, caspase‐1, ASC, and GAPDH, with an initial denaturation step at 95°C for 30 s. Primer sequences can be found in Table 1. The fold change was determined using the 2^−ΔΔCt^ method, with GAPDH serving as the endogenous reference gene for NLRP3, IL‐18, IL‐1β, and caspase‐1.

**Table 1:** Primer sequences.

Gene	Sequence (5^′^–3^′^)
NLRP3 F (mouse)	TTATTTGTACCCAAGGCTGCT
NLRP3 R (mouse)	GGCTTAGGTCCACACAGAAA
IL‐18 F (mouse)	TGCCACCTTTTGACAGTGATG
IL‐18 R (mouse)	AAGGTCCACGGGAAAGACAC
Caspase‐1 F (mouse)	CTCGTACACGTCTTGCCCTC
Caspase‐1 R (mouse)	CCTCTTTCACCATCTCCAGAGC
ASC F (mouse)	GAGTACAGCCAGAACAGGACACT
ASC R (mouse)	ACTGCCATGCAAAGCATCCA
GAPDH F (mouse)	GGGGTCGTTGATGGCAACA
GAPDH R (mouse)	AGGTCGGTGTGAACGGATTTG
NLRP3 F (human)	CTGGCATCTGGGGAAACCT
NLRP3 R (human)	TCCTTAGGCTTCGGTCCACA
IL‐18 F (human)	TGCAGTCTACACAGCTTCGG
IL‐18 R (human)	GCAGCCATCTTTATTCCTGCG
Caspase‐1 F (human)	GCAGCCATCTTTATTCCTGCG
Caspase‐1 R (human)	TGTACCTTCACCCATGGAACG
ASC F (human)	TCTACCTGGAGACCTACGGC
ASC R (human)	TCCAGAGCCCTGGTGC
GAPDH F (human)	GAAAGCCTGCCGGTGACTAA
GAPDH R (human)	GCCCAATACGACCAAATCAGAG

### 2.22. Molecular Docking

The protein structure of NLRP3 (ID: 8WSM) was downloaded from the Protein Data Bank (PDB) database (https://www.rcsb.org/). These compound structures were obtained from PubChem in SDF format (https://pubchem.ncbi.nlm.nih.gov/). The protein and compound structures were imported into Discovery Studio 2019, followed by the removal of all water molecules from the protein crystal structure and subsequent hydrogenation treatment. The amino acid residues within a 10 Å radius around the cocrystallized ligand were defined in the crystal structure as binding pockets. Preprocessing of the protein structure, ligand energy minimization, ligand structure preparation, and molecular docking were all performed using default values.

### 2.23. Statistical Analysis

All data were expressed as mean ± SD and analyzed with SPSS 25.0 software. To assess treatment effects, a one‐way ANOVA and a paired *T*‐test were employed. For multiple comparisons, Duncan’s tests were utilized. A *p*‐value < 0.05 was considered statistically significant.

## 3. Results

### 3.1. The Composition Analysis of Flavonoids From SYQ

First, we identified the chemical compounds of SYQ flavonoids by HPLC. The standard curves of procyanidin B1, catechin, kaempferol 3‐O‐glucoside‐7‐O‐rhamnoside, kaempferol‐3‐O‐rutinoside, and astragalin were *y* = 12.694x + 0.2947 (*R* ^2^ = 0.9996), *y* = 17.208x + 3.0752 (*R* ^2^ = 0.9998), *y* = 400.36x + 2.0409 (*R* ^2^ = 0.9999), *y* = 109.22x + 3.0752 (*R* ^2^ = 0.9998), and *y* = 241.01x + 6.1943 (*R* ^2^ = 0.9993). The five compounds in SYQ flavonoids were 25.94 ± 3.21, 102.25 ± 3.84, 7.31 ± 1.14, 4.07 ± 0.56, and 3.06 ± 0.11 mg/g, respectively (Figure 1).

Figure 1HPLC profile of standards and SYQ flavonoids. (A) Standards of procyanidin B1, catechin, kaempferol 3–0‐glucoside‐7‐O‐rhamnoside, kaempferol‐3‐O‐rutinoside, and astragalin and (B) the sample of SYQ flavonoids were measured for three times by HPLC. The active compounds of SYQ for ALI treatment.

**Figure:**
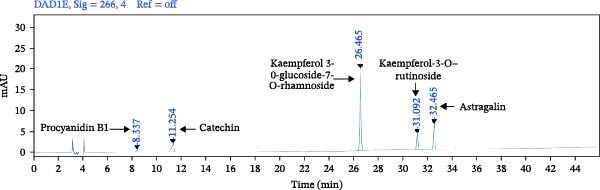
(A)

**Figure:**
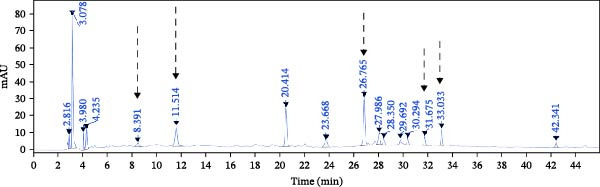
(B)

### 3.2. Prediction of the Active Ingredients of SYQ Flavonoids and Its Potential Mechanism on ALI by Network Pharmacology

In the GSE32707 and GSE2322 datasets, 7351 and 934 DEGs were identified, respectively (Figure 1A). Additionally, 36 active compounds and 179 target genes of SYQ flavonoids were extracted from relevant databases (Supporting Information 1: Table S2). By intersecting the ALI‐related DEGs with the SYQ flavonoid target genes, 11 overlapping genes were pinpointed (Figure 2B). To further elucidate interactions, a TCM‐compound‐target‐disease network was constructed (Figure 2C), revealing that procyanidin B1 (MOL000004), quercetin (MOL000098), isorhamnetin (MOL000354), kaempferol (MOL000422), catechin (MOL000492), isovitexin (MOL002322), and kaempferide (MOL004564) were predicted as key bioactive compounds of SYQ for ALI treatment.

Figure 2Network pharmacology analysis to predict the active compounds of SYQ and its mechanism for ALI treatment. (A) The volcano diagram of GSE2322 and GSE32707. (B) Venn diagram of the targets of GSE2322, GSE32707, and SYQ. (C) TCM‐compound‐target‐disease network. (D) PPI network of the intersection targets. (E) GO function, including BP, CC and MF, and KEGG enrichment analysis, was performed.

**Figure:**
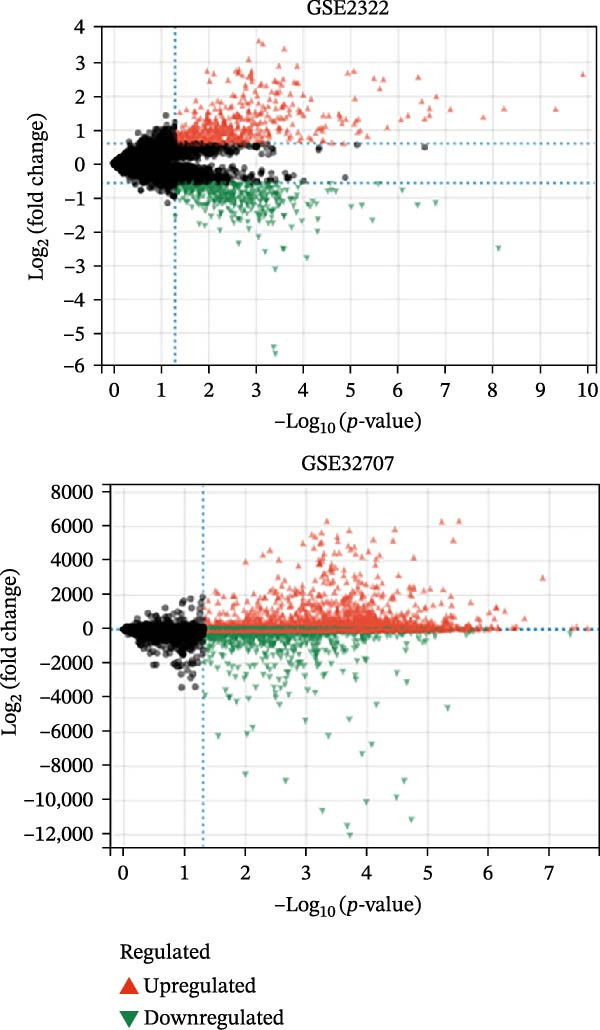
(A)

**Figure:**
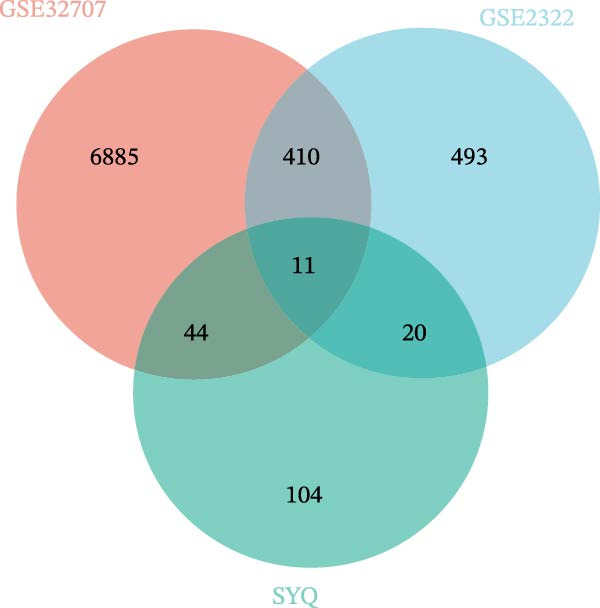
(B)

**Figure:**
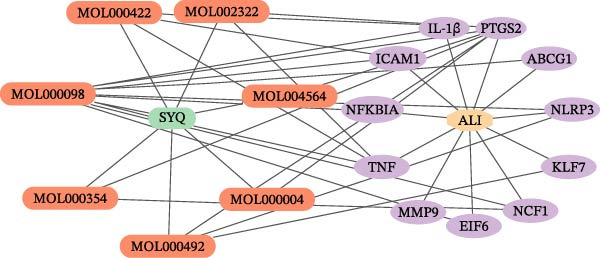
(C)

**Figure:**
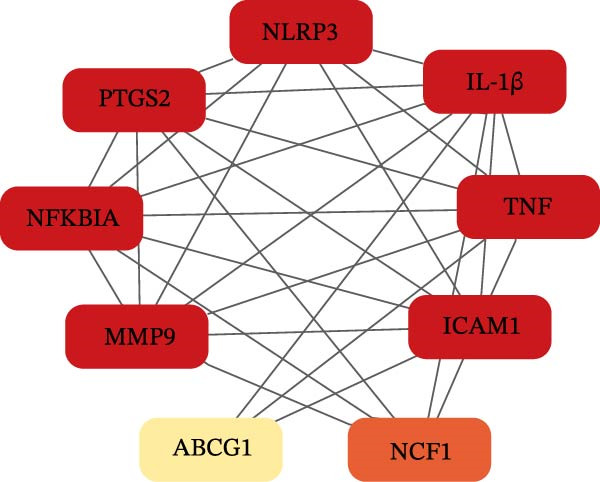
(D)

**Figure:**
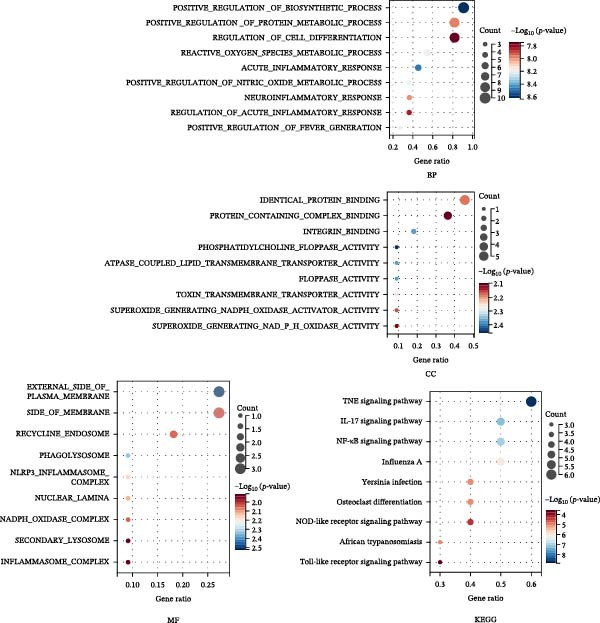
(E)

The PPI analysis identified IL‐1β, NLRP3, TNF, ICAM, and PTGS2 as the top five hub proteins based on their degree values, providing critical clues to the underlying therapeutic targets and mechanistic pathways (Figure 2D). GO functional enrichment analysis indicated that these targets were primarily involved in BP such as positive regulation of biosynthetic processes and acute inflammatory responses; cellular components (CC) including identical protein binding and protein‐containing complexes; and molecular functions (MFs) associated with NLRP3 inflammasome complex formation. KEGG pathway enrichment analysis further highlighted that these targets were significantly enriched in inflammatory signaling pathways, including the NOD‐like receptor, TNF, and IL‐17 signaling pathways (Figure 2E).

### 3.3. The Protect Effect of Total Flavonoids From SYQ on ALI

HE staining (Figure 3A) revealed intact lung structure in the control group, characterized by thin bronchial walls, uniform lumen thickness, and preserved alveolar architecture. In contrast, the model group exhibited marked tracheal wall thickening and lumen narrowing. SYQ‐F treatment alleviated both luminal wall thickening and lumen constriction, a trend consistent with Figure 3C. Pathological scores of lung inflammation were significantly higher in the model group versus the control group (*p* < 0.05, 0.01) but reduced in the SYQ‐FH, FM, FL, and MCC950 groups.

Figure 3SYQ‐F alleviated LPS‐induced ALI in mice. (A) HE staining of lung tissue (200×). (B) Masson staining of lung tissue (200×). (C) Quantification of the lung injury score. (D) The lung wet/dry (W/D) weight ratio. (E–K) ELISA detected the levels of IL‐1β, IL‐6, and TNF‐α in BALF and serum, as well as the IL‐10 in BALF. (L) LDH levels in BALF. ^##^ *p* < 0.01, ^#^ *p* < 0.05 vs. control group; ^$$^ *p* < 0.01, ^$^ *p* < 0.05 vs. model group.

**Figure:**
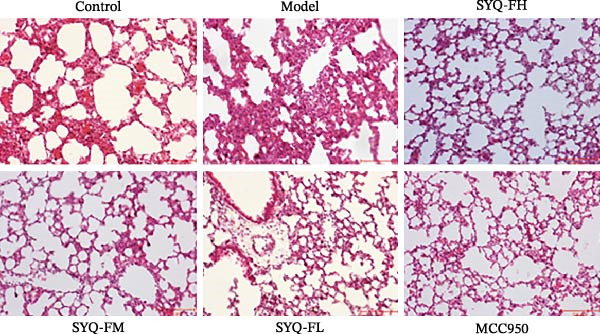
(A)

**Figure:**
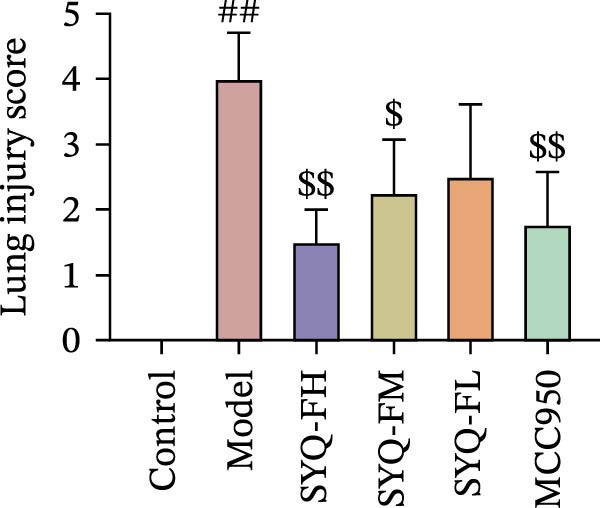
(B)

**Figure:**
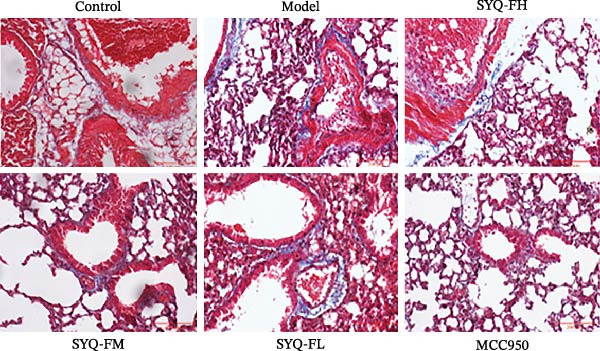
(C)

**Figure:**
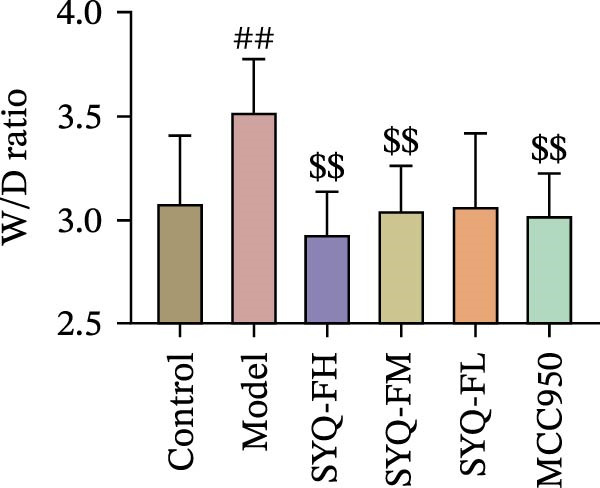
(D)

**Figure:**
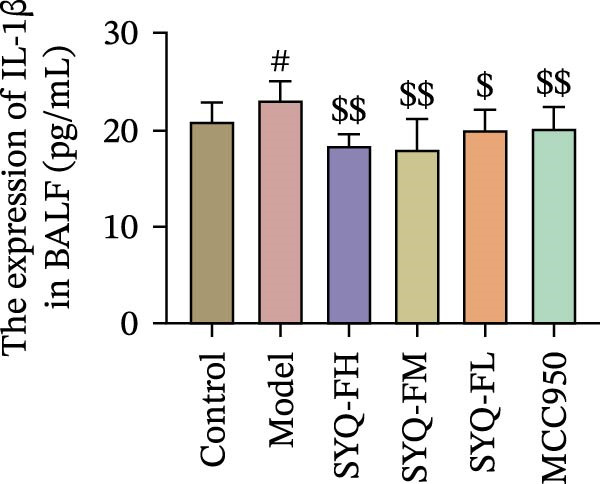
(E)

**Figure:**
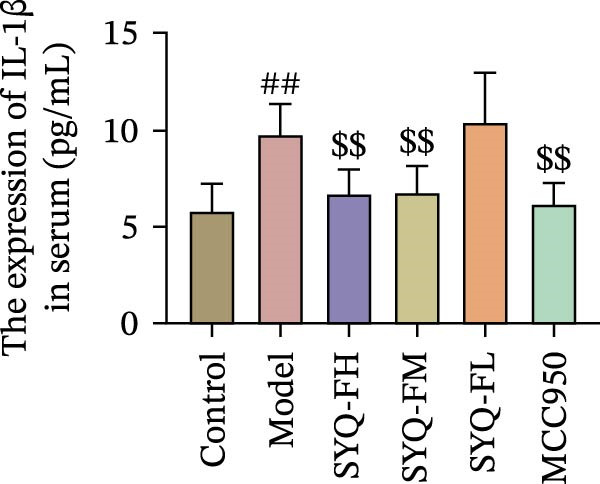
(F)

**Figure:**
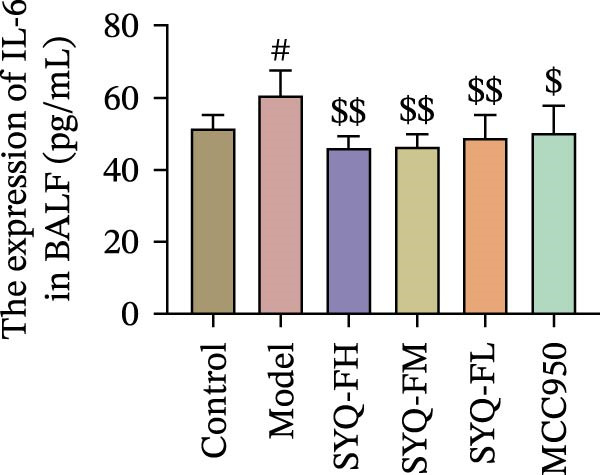
(G)

**Figure:**
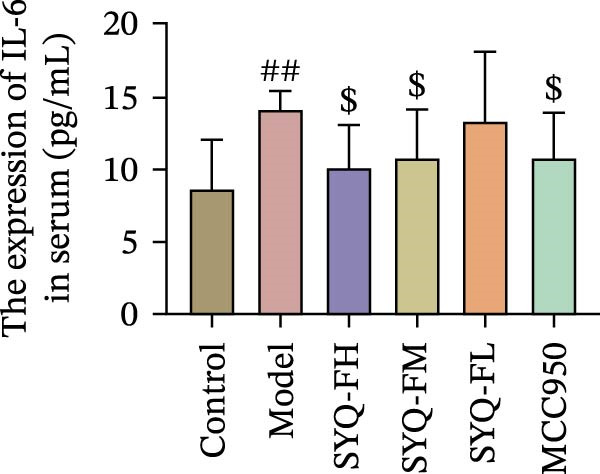
(H)

**Figure:**
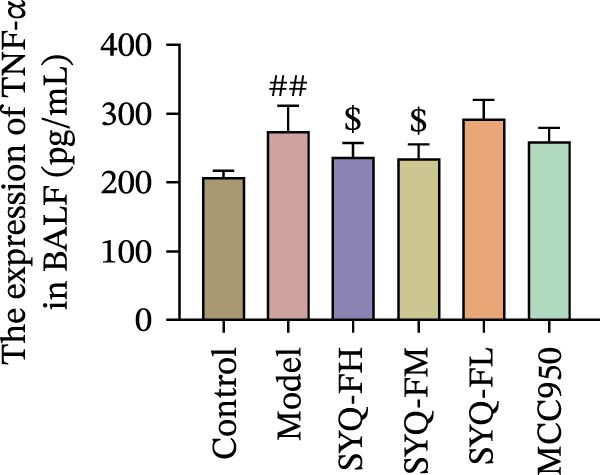
(I)

**Figure:**
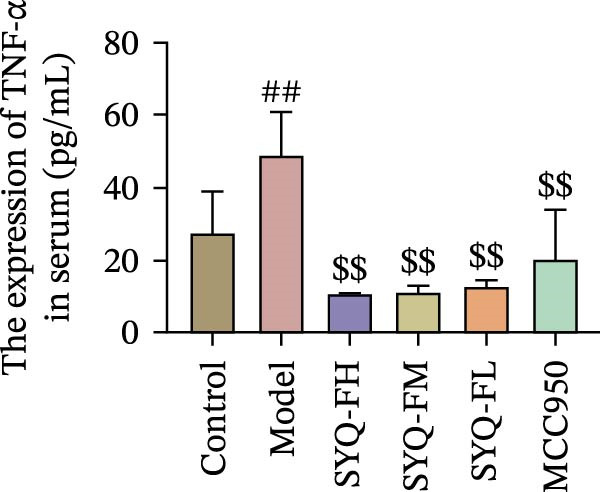
(J)

**Figure:**
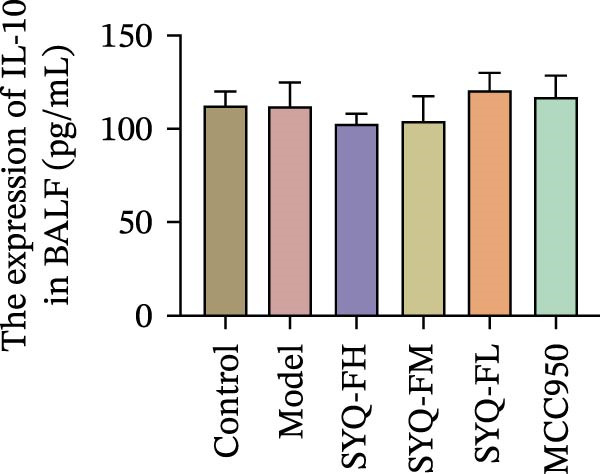
(K)

**Figure:**
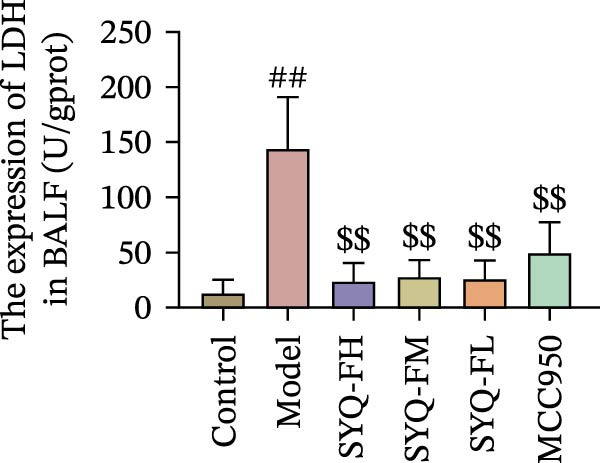
(L)

Masson staining (Figure 3B) showed LPS‐induced collagen deposition in model lungs, which was mitigated by SYQ‐F. Elevated lung W/D ratios (indicating pulmonary edema) in the model group were significantly reduced by high and medium doses of SYQ‐F (*p* < 0.01).

Consistent with bioinformatic predictions, the anti‐inflammatory role of SYQ flavonoids and the pro‐inflammatory cytokines IL‐1β, IL‐6, and TNF‐α were markedly upregulated in the model serum/BALF (*p* < 0.05, 0.01 vs. control) but suppressed by SYQ‐F treatment. Anti‐inflammatory IL‐10 levels showed no intergroup differences (Figure 3E–J). Additionally, model LDH levels were reduced by SYQ‐FH, FM, and FL groups (*p* < 0.01, Figure 3L). Collectively, these data demonstrate that SYQ total flavonoids alleviate LPS‐induced lung injury by suppressing inflammation.

### 3.4. SYQ Flavonoids Regulated the NLRP3 Signaling Way In Vivo and Vitro Experiment

Network pharmacology analysis highlighted a tight correlation between SYQ flavonoid compounds and inflammatory pathways in ALI treatment, with NLRP3 and its downstream pathways as key nodes. To validate this, we used WB and qRT‐PCR to measure NLRP3 signaling components. qRT‐PCR revealed upregulated NLRP3, caspase‐1, ASC, and IL‐18 mRNA in the model group versus controls (*p* < 0.05, 0.01), which were downregulated by SYQ‐F and MCC950 (Figure 4A). WB confirmed elevated protein levels of NLRP3, cleaved‐caspase‐1, ASC, IL‐18, and IL‐1β post‐LPS, while the SYQ‐F and MCC950 groups showed significant reductions (*p*  < 0.05, 0.01, Figure 4B). These data indicate SYQ flavonoids inhibit NLRP3 signaling.

Figure 4SYQ‐F inhibited NLRP3 signaling pathway in the LPS‐induced ALI. (A) qRT‐PCR was used to measure the levels of NLRP3, caspase 1, ASC, and IL‐18 mRNA. (B) WB was used to evaluate the expressions of NLRP3, cleaved‐caspase 1, ASC, IL‐18, and IL‐1β. ^##^ *p* < 0.01, ^#^ *p* < 0.05 vs. control group; ^$$^ *p* < 0.01, ^$^ *p* < 0.05 vs. model group. (C) qRT‐PCR was used to detect the levels of NLRP3, ASC, IL‐18, IL‐1β, and caspase‐1 mRNAs. (D) The expressions of NLRP3, IL‐18, and IL‐1β were measured by WB. (E, F) Immunofluorescence assays were used to determine the expression of ASC and cleaved‐caspase‐1. ^##^ *p* < 0.01 vs. control group; ^$$^ *p* < 0.01, ^$^ *p* < 0.01 vs. model group.

**Figure:**
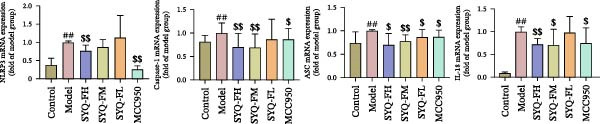
(A)

**Figure:**
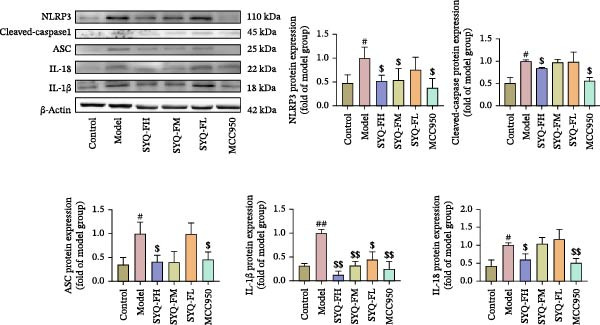
(B)

**Figure:**
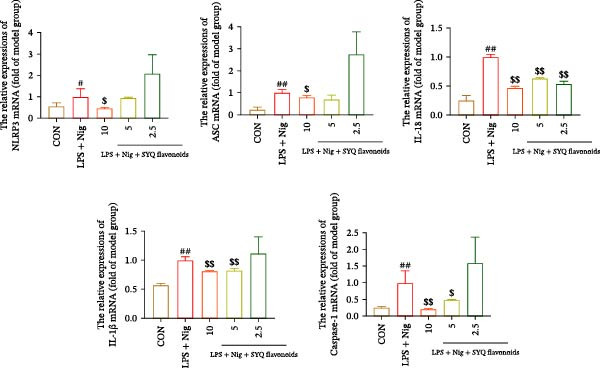
(C)

**Figure:**
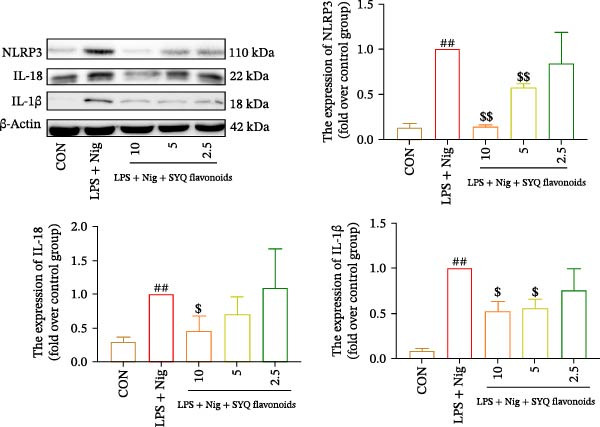
(D)

**Figure:**
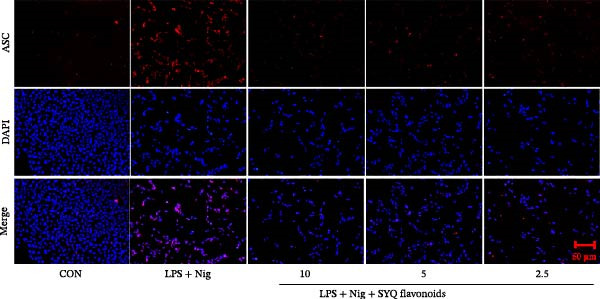
(E)

**Figure:**
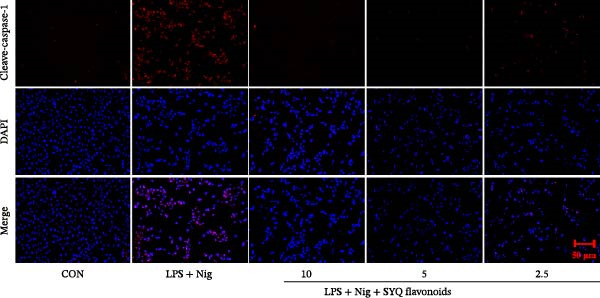
(F)

To further assess SYQ regulatory effects on NLRP3 inflammasome activation, we induced NLRP3 activation with LPS + nigericin and evaluated NLRP3, ASC, IL‐1β, IL‐18, and caspase‐1 via qRT‐PCR, WB, and immunofluorescence. LPS + nigericin stimulation significantly upregulated both mRNA and protein levels of these factors, an effect reversed by SYQ flavonoids (Figure 4C–F, *p*  < 0.05, 0.01). Collectively, these findings confirm that SYQ flavonoids ameliorate ALI by suppressing NLRP3 inflammasome activation.

### 3.5. SYQ Flavonoids Improved LPS + Nigericin‐Induced Cell Viability

To further investigate how SYQ flavonoids regulate NLRP3 inflammasome activation, we induced NLRP3 activation using LPS + nigericin. First, we confirmed SYQ flavonoids (10–0.078 μg/mL) were nontoxic to BEAS‐2B cells (Figure 5A, *p*  > 0.05). Post‐LPS/nigericin induction, SYQ flavonoids dose‐dependently restored cell viability: from 38.7% to 46.5% (2.5 μg/mL), 52.1% (5 μg/mL), and 61.7% (10 μg/mL) (*p* < 0.01; Figure 5B).

Figure 5SYQ‐F attenuates LPS + nigericin‐induced ALI by preserving mitochondrial function and reducing ROS. (A,B) MTT assay for cell viability detection. JC‐1 assay: (C) fluorescence microplate quantification and (E) fluorescence microscopy imaging of mitochondrial membrane potential changes. DCFH‐DA assay: (D) fluorescence microplate quantification and (F) fluorescence microscopy imaging of intracellular ROS levels. ^##^ *p* < 0.01 vs. control group; ^$$^ *p* < 0.01 vs. LPS + Nig group.

**Figure:**
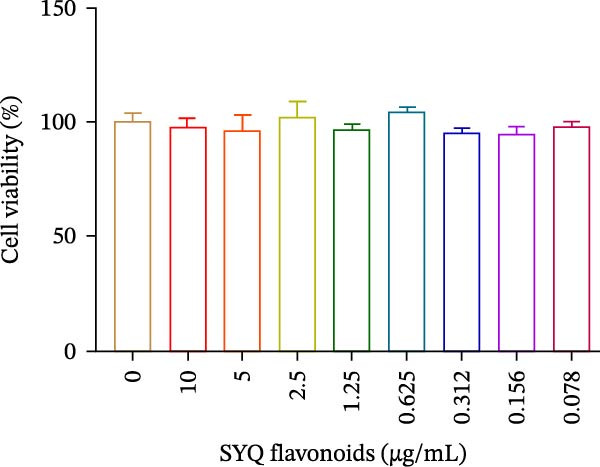
(A)

**Figure:**
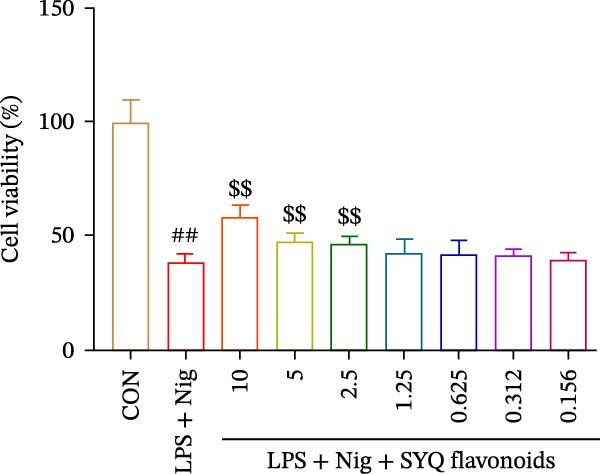
(B)

**Figure:**
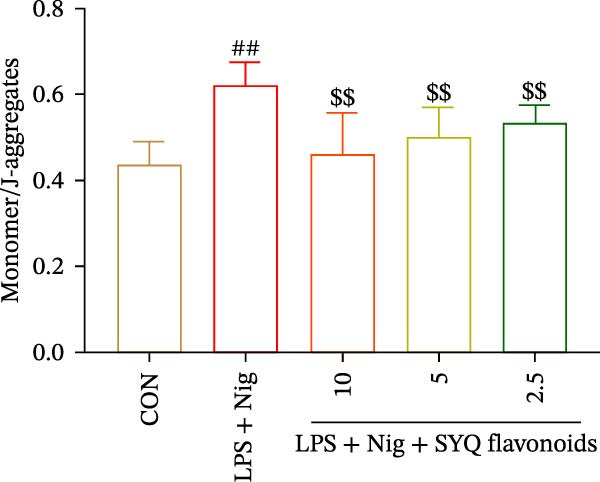
(C)

**Figure:**
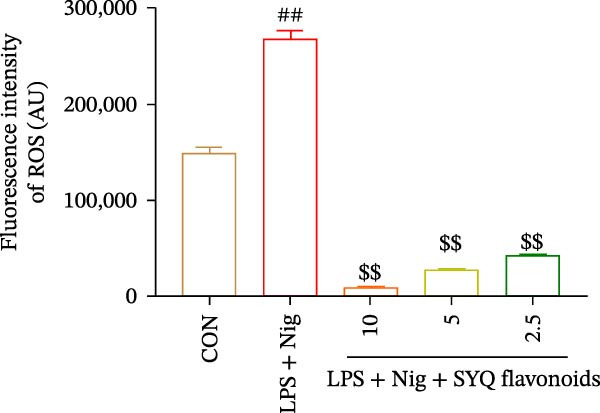
(D)

**Figure:**
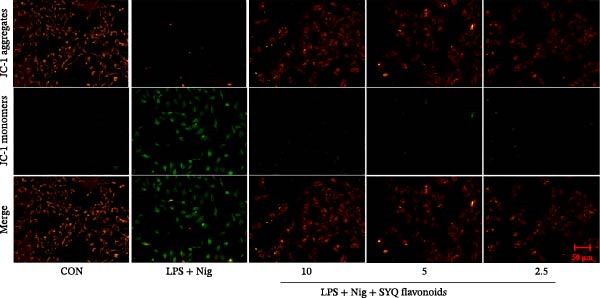
(E)

**Figure:**
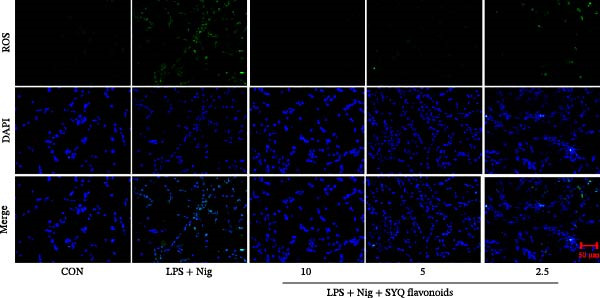
(F)

JC‐1 assays showed SYQ flavonoids dose‐dependently reversed LPS/nigericin‐disrupted mitochondrial membrane potential. The green/red fluorescence ratio decreased from 0.62 ± 0.05 to 0.53 ± 0.04 (2.5 μg/mL), 0.50 ± 0.06 (5 μg/mL), and 0.46 ± 0.09 (10 μg/mL) (*p* < 0.01; Figure 5C,E), indicating improved mitochondrial function with higher doses.

Consistently, SYQ flavonoids dose‐dependently suppressed ROS overproduction. Fluorescence intensity dropped from 268053 ± 9378 to 42608 ± 850 (2.5 μg/mL, *p*  < 0.05), 27523 ± 2054 (5 μg/mL, *p*  < 0.01), and 9046 ± 438 (10 μg/mL, *p*  < 0.01) (Figure 5D–F), reflecting enhanced antioxidant capacity.

### 3.6. Effect of SYQ Flavonoids on IL‐1β and IL‐18 Production in LPS + Nigericin‐Induced ALI In Vitro After NLRP3 Knocked Down

To further validate the dependency of phenotypic changes on NLRP3, we evaluated cell viability and cytotoxicity in siRNA‐transfected cells. As shown in Figure 6A,B; LPS + nigericin significantly reduced viability and increased LDH release in siNC cells (*p* < 0.01), effects markedly rescued by SYQ‐F (*p* < 0.01). Conversely, NLRP3 knockdown (siNLRP3) alone conferred cytoprotection, and SYQ‐F provided no additional benefit in siNLRP3 cells (*p* > 0.05).

Figure 6SYQ‐F The impact of SYQ flavonoids on IL‐18 and IL‐1β in BEAS‐2B cells stimulated with LPS and nigericin after NLRP3 knockdown. (A) MTT assay was used to measure the cell viability. (B) LDH release was measured. (C) The expressions of NLRP3, IL‐18, and IL‐1β were measured by WB. ##*p* < 0.01.

**Figure:**
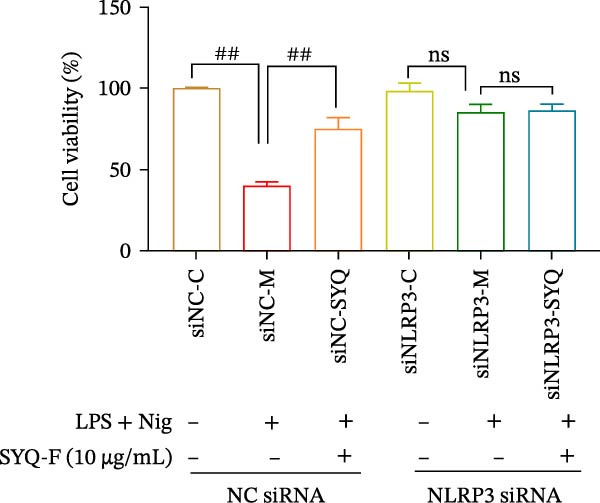
(A)

**Figure:**
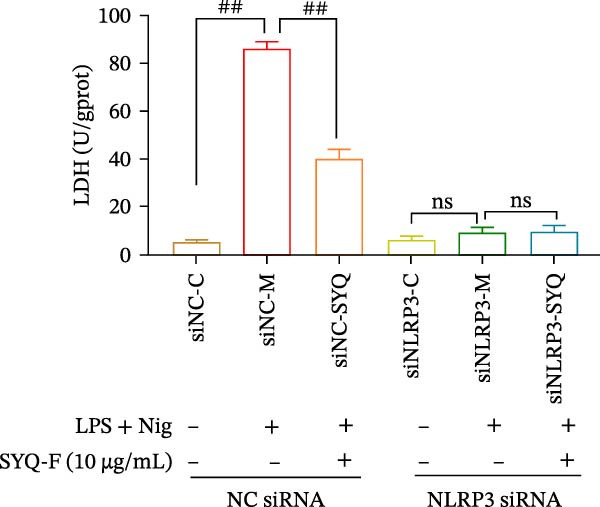
(B)

**Figure:**
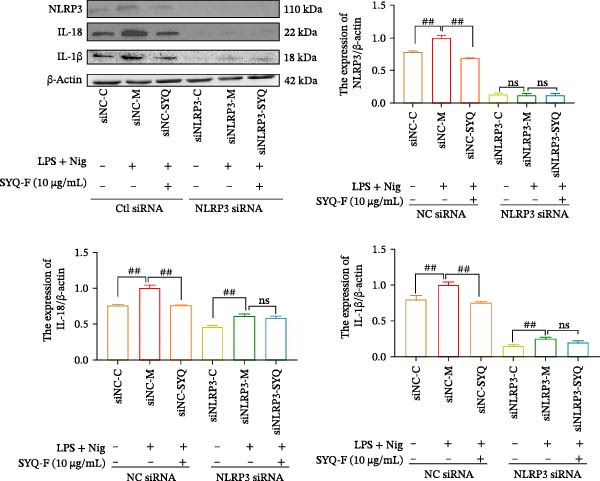
(C)

We next used WB to assess IL‐1β and IL‐18 expression following LPS + nigericin induction in NLRP3‐knockdown cells treated with SYQ flavonoids (Figure 6C). Compared to siNC‐C, siNC‐M (LPS + nigericin‐treated siNC cells) showed significantly upregulated NLRP3, IL‐18, and IL‐1β (*p* < 0.01); this upregulation was markedly attenuated by SYQ‐F (siNC‐SYQ group, *p*  < 0.01). In siNLRP3 cells, despite LPS + nigericin stimulation (siNLRP3‐M), IL‐18 and IL‐1β levels remained elevated (vs. siNLRP3‐C, *p*  < 0.01), though NLRP3 expression was unchanged. Notably, SYQ‐F treatment (siNLRP3‐SYQ) did not further alter IL‐18/IL‐1β levels compared to siNLRP3‐M (*p* > 0.05). These results indicate that the inhibitory effects of SYQ flavonoids on LPS + nigericin‐induced IL‐1β/IL‐18 secretion depend on NLRP3, supporting the notion that their therapeutic action against ALI involves suppressing NLRP3 inflammasome activation.

### 3.7. Molecular Docking Results

Integrating HPLC profiling of SYQ flavonoids with network pharmacology predictions of ALI‐active components, we identified catechin and procyanidin B1 as key therapeutic candidates. Combined with network‐based target prediction and in vivo/in vitro validation, NLRP3 was highlighted as a critical target of SYQ flavonoids for ALI. To further evaluate their binding affinity, molecular docking was performed between these compounds and NLRP3 (the molecular docking results of other main components of SYQ with NLRP3 were in the Supporting Information 1: Figure S1).

3D structural analysis revealed strong interactions: catechin formed three conventional hydrogen bonds (with THR439, ARG578, and ARG351), two π‐alkyl interactions (ILE411and ALA227), one π‐sulfur bond (MET408), and one π‐π T‐shaped interaction (PHE575) with NLRP3 (Figure 7A). Procyanidin B1 engaged in three conventional hydrogen bonds, two π‐alkyl, and one π‐π stacked interaction with NLRP3 (Figure 7B).

Figure 7The molecular docking images of NLRP3 and SYQ representative compounds. 2D and 3D images and zoom in on specific areas of NLRP3 with (A) catechin and (B) procyanidin B1.

**Figure:**
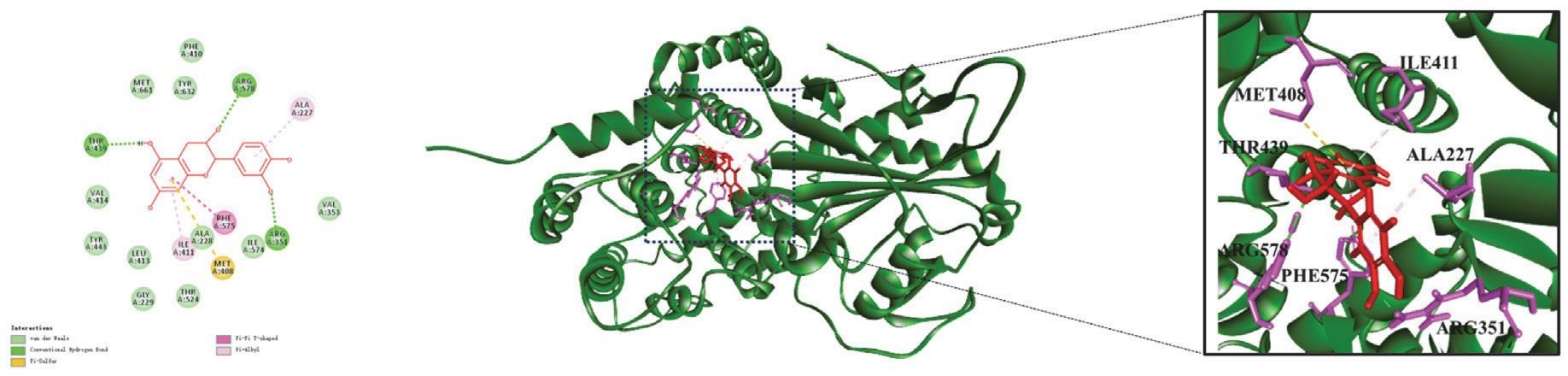
(A)

**Figure:**
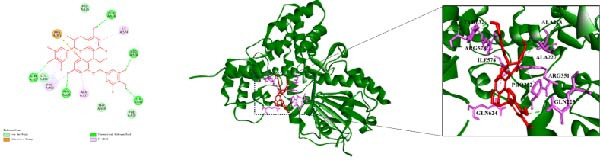
(B)

Binding energy (threshold: <−5 kcal/mol for significant activity) further supported these interactions (Table 2): both compounds exhibited low binding energies, indicating strong affinity for NLRP3. These results suggest that SYQ flavonoid representatives (catechin and procyanidin B1) bind effectively to NLRP3, underscoring its pivotal role in SYQ‐mediated ALI treatment.

**Table 2:** The binding energy results (kcal/mol).

Target	Compounds	Binding energy
NLRP3	Catechin	−7.60
	Procyanidin B1	−12.73

## 4. Discussion

ALI is a life‐threatening inflammatory syndrome with limited therapeutic options [2]. While the NLRP3 inflammasome has emerged as a key therapeutic target, existing inhibitors face challenges such as incomplete pathway coverage and systemic side effects [13]. *Tetrastigma hemsleyanum* Diels et Gilg (SYQ) presents a novel multitarget strategy, distinguishing itself from single‐target agents by modulating both the priming and activation phases of inflammation.

Using network pharmacology, we first identified seven potential bioactive flavonoid compounds in SYQ that may contribute to its ALI therapeutic effects. HPLC analysis further highlighted catechin and procyanidin B1 as likely key contributors to its protective effects in this study. Molecular docking confirmed robust interactions between these flavonoids and NLRP3: catechin exhibited a binding energy of −7.60 kcal/mol, while procyanidin B1 reached −12.73 kcal/mol—both significantly surpassing the biologically relevant binding threshold (<−5 kcal/mol) [14], strongly suggesting that both compounds can effectively engage the NLRP3 target. These compounds exhibit drug‐like binding stability through multiple hydrogen bonds and hydrophobic interactions. These values, combined with multiple hydrogen bonds and hydrophobic interactions, suggest strong target engagement and drug‐like binding stability.

The therapeutic potential for ALI of catechin is supported by prior studies demonstrating its roles in inhibiting RasGRP1 [15] and regulating the miR‐182/GGPPS1 pathway [16]. Procyanidin B1, a major bioactive component of SYQ, has shown anti‐inflammatory and neuroprotective effects in neuroinflammation models [17] and anti‐lung cancer activity [18]. Our findings extend this profile, suggesting procyanidin B1 may also serve as a key therapeutic agent for ALI. Collectively, these data propose catechin and procyanidin B1 as the primary pharmacological basis for SYQ flavonoids’ ALI treatment effects.

The NLRP3 inflammasome is a central mediator of ALI pathogenesis: LPS‐induced activation upregulates NLRP3, ASC, and caspase‐1, ultimately triggering IL‐1β and IL‐18 release and driving ALI [19]. PPI and KEGG pathway analyses further implicated NLRP3, IL‐1β, TNF, and the NOD‐like receptor signaling pathway as critical to SYQ flavonoids’ therapeutic effects. In vivo validation in an ALI mouse model confirmed that SYQ flavonoids downregulate NLRP3 inflammasome activation, suppressing IL‐1β, IL‐18, and cleaved caspase‐1. This aligns with prior reports that IL‐1β and IL‐18 levels in BALF and lung tissue are elevated in LPS‐induced ALI, correlating with enhanced NLRP3 and caspase‐1 activity [20].

To further clarify SYQ flavonoids’ modulation of the NLRP3 inflammasome, we activated the inflammasome in vitro using LPS + nigericin. The results showed that SYQ flavonoids significantly attenuated ALI by reducing NLRP3, ASC, IL‐1β, and IL‐18 expression. Critically, siRNA‐mediated NLRP3 knockdown abolished SYQ‐F’s protective effects: MTT/LDH data revealed loss of cell viability protection and reduced IL‐1β/IL‐18 secretion in NLRP3‐deficient cells, demonstrating target specificity. This mirrors findings by He et al. [21], where NLRP3 knockdown reduced KA‐induced neuronal loss and inflammation, with curcumin failing to exert additional inhibition—an effect attributed to its complete dependence on the NLRP3 pathway. Together, these results indicate that SYQ flavonoids likely exert their ALI‐protective effects by specifically targeting the NLRP3 inflammasome, thereby suppressing pro‐inflammatory cytokine production (e.g., IL‐1β and IL‐18).

As a traditional remedy for pneumonia and fever [22], SYQ has long been used empirically. Our study validates its efficacy in cellular and animal ALI models, providing experimental support for expanding SYQ‐derived therapies into clinical applications for inflammatory lung diseases.

## 5. Conclusion

In conclusion, our findings underscore the potential of SYQ flavonoids as promising natural therapeutic candidates for ALI treatment, which exhibit significant therapeutic effects on ALI by specifically binding to the NLRP3 inflammasome. This conclusion provides a robust scientific foundation for developing new natural product‐based therapies and paves the way for further research and potential clinical applications.

## Author Contributions

Lianghui Zhan wrote the manuscript. Lingling Li and Lianghui Zhan performed the experiment. Xuechun Jiang and Lianghui Zhan made the bioinformation analysis. Xuechun Jiang, Xiaojun Wu, and Jie Zhou participated in data analysis. Jinbao Pu and Weiqing Liang designed the study and provided the financial support. Sheng Zhu and Changcheng Shu provided the stable quality of SYQ for this experiment.

## Funding

This work was the supported by the Zhejiang TCM Science and Technology Plan (Grant 2023ZR076), the National Natural Science Foundation of China (Grant 82505217), the Zhejiang Medicine and Health Science and Technology Project (Grant 2024KY877), the Quzhou Science and Technology Plan Project (Grant 2023K092), the Zhejiang Traditional Chinese Medicine Science and Technology Planning Project (Grant 2020ZX004), the Zhejiang Key Discipline in Traditional Chinese Medicine for Pharmaceutical Botony (Grant 2024‐XK‐06), the Zhejiang TCM Science and Technology Plan (Grant 2024ZR008), and the Project on the Sustainable Utilization Capacity of Precious Traditional Chinese Medicinal Resources (Grant 2060302).

## Disclosure

This manuscript has not been published or presented elsewhere in part or in entirety and is not under consideration by another journal. We have read and understood your journal’s policies, and we believe that neither the manuscript nor the study violates any of these.

## Ethics Statement

All animal procedures were ethically approved by the Animal Ethics Community of Zhejiang Academy of Traditional Chinese Medicine (Ethical Number: 2023‐068), in compliance with European Community Guidelines for Laboratory Animal Care.

## Consent

The authors have nothing to report.

## Conflicts of Interest

The authors declare no conflicts of interest.

## Supporting Information

Additional supporting information can be found online in the Supporting Information section.

## Supporting information


**Supporting Information** Table S1 presents the grading and scoring criteria for assessing inflammatory pathology in lung tissue. Table S2 lists the flavonoids contained in SYQ and their corresponding targets. Figure S1 showed that siRNA‐mediated interference led to a marked reduction in NLRP3 protein levels. Figure S2 displays the molecular docking results between NLRP3 and three flavonoid compounds: kaempferol 3‐O‐glucoside‐7‐O‐rhamnoside, kaempferol‐3‐O‐rutinoside, and astragalin.

## Data Availability

The data generated in this study are available from the corresponding author upon request.
