# Supplementary material for: Tetrastigma hemsleyanum Diels et Gilg Flavonoids Against Acute Lung Injury Via Block NLRP3 Inflammation
Source: Mediators Inflamm. 2026 Mar 8;2026:2279402. doi: 10.1155/mi/2279402 (PMC12968339; doi:10.1155/mi/2279402)
Supplement: Supplementary file 1 — Supporting Information Table S1 presents the grading and scoring criteria for assessing inflammatory pathology in lung tissue. Table S2 lists the flavonoids contained in SYQ and their corresponding targets. Figure S1 showed that siRNA‐mediated interference led to a marked reduction in NLRP3 protein levels. Figure S2 displays the molecular docking results between NLRP3 and three flavonoid compounds: kaempferol 3‐O‐glucoside‐7‐O‐rhamnoside, kaempferol‐3‐O‐rutinoside, and astragalin. [file MI-2026-2279402-s001.docx]

**Supplementary table.1** Grading and scoring standard of inflammatory pathology of lung tissue

| Pathology grading score | Increased numbers of blood vessels and eosinophils in the surrounding area | Edema | Epithelial cell injury |
| --- | --- | --- | --- |
| 0 | Normal | Normal | Normal |
| 1 | Mild cellular infiltration with no tissue damage | Mild diffuse edema | Mild cellular injury |
| 2 | Moderate cellular infiltration, mild tissue damage | Mild alveolar and bronchiolar edema | Mild cellular injury |
| 3 | Moderate cellular infiltration and moderate tissue damage | Regional and focal edema | Moderate cellular injury |
| 4 | Severe cellular infiltration, evident tissue damage | Obvious edema | Moderate cellular injury |
| 5 | Severe cellular infiltration, evident tissue damage | Pulmonary edema resembling pneumonia | Epithelial deformation and mucosal cell proliferation |

**Supplementary table.2** The compounds of SYQ flavonoids and the targets

| MOL | Compounds | Targets |
| --- | --- | --- |
| MOL004564 | kaempferide | NOS2 |
| MOL004564 | kaempferide | PTGS1 |
| MOL004564 | kaempferide | ESR1 |
| MOL004564 | kaempferide | AR |
| MOL004564 | kaempferide | PPARG |
| MOL004564 | kaempferide | PTGS2 |
| MOL004564 | kaempferide | DPP4 |
| MOL004564 | kaempferide | MAPK14 |
| MOL004564 | kaempferide | GSK3B |
| MOL004564 | kaempferide | HSP90 |
| MOL004564 | kaempferide | NOS2 |
| MOL004564 | kaempferide | PTGS1 |
| MOL004564 | kaempferide | ESR1 |
| MOL004564 | kaempferide | AR |
| MOL004564 | kaempferide | PPARG |
| MOL004564 | kaempferide | PTGS2 |
| MOL004564 | kaempferide | DPP4 |
| MOL004564 | kaempferide | MAPK14 |
| MOL004564 | kaempferide | GSK3B |
| MOL004564 | kaempferide | HSP90 |
| MOL000004 | procyanidin B1 | PTGS1 |
| MOL000004 | procyanidin B1 | SCN5A |
| MOL000004 | procyanidin B1 | PTGS2 |
| MOL000004 | procyanidin B1 | NOS3 |
| MOL000004 | procyanidin B1 | GABRA1 |
| MOL000004 | procyanidin B1 | HSP90 |
| MOL000004 | procyanidin B1 | PIK3CG |
| MOL000004 | procyanidin B1 | PRKACA |
| MOL000004 | procyanidin B1 | IGHG1 |
| MOL000004 | procyanidin B1 | NCOA2 |
| MOL000004 | procyanidin B1 | PKIA |
| MOL000492 | Catechin | PTGS1 |
| MOL000492 | Catechin | ESR1 |
| MOL000492 | Catechin | PTGS2 |
| MOL000492 | Catechin | HSP90 |
| MOL000492 | Catechin | DPEP1 |
| MOL000492 | Catechin | PRKACA |
| MOL000492 | Catechin | NCOA2 |
| MOL000492 | Catechin | CAMKK2 |
| MOL000492 | Catechin | FASN |
| MOL000492 | Catechin | PPARG |
| MOL000492 | Catechin | NLRP3 |
| MOL000492 | Catechin | KLF7 |
| MOL000354 | isorhamnetin | NOS2 |
| MOL000354 | isorhamnetin | PTGS1 |
| MOL000354 | isorhamnetin | ESR1 |
| MOL000354 | isorhamnetin | AR |
| MOL000354 | isorhamnetin | PPARG |
| MOL000354 | isorhamnetin | PTGS2 |
| MOL000354 | isorhamnetin | PTPN1 |
| MOL000354 | isorhamnetin | ESR2 |
| MOL000354 | isorhamnetin | DPP4 |
| MOL000354 | isorhamnetin | MAPK14 |
| MOL000354 | isorhamnetin | GSK3B |
| MOL000354 | isorhamnetin | HSP90 |
| MOL000354 | isorhamnetin | CDK13 |
| MOL000354 | isorhamnetin | PIK3CG |
| MOL000354 | isorhamnetin | PRKACA |
| MOL000354 | isorhamnetin | PRSS1 |
| MOL000354 | isorhamnetin | PIM1 |
| MOL000354 | isorhamnetin | CCNA2 |
| MOL000354 | isorhamnetin | NCOA2 |
| MOL000354 | isorhamnetin | CAMKK2 |
| MOL000354 | isorhamnetin | PYGM |
| MOL000354 | isorhamnetin | PPARD |
| MOL000354 | isorhamnetin | CHEK1 |
| MOL000354 | isorhamnetin | AKR1B10 |
| MOL000354 | isorhamnetin | NCOA1 |
| MOL000354 | isorhamnetin | F7 |
| MOL000354 | isorhamnetin | F2 |
| MOL000354 | isorhamnetin | NOS3 |
| MOL000354 | isorhamnetin | ACHE |
| MOL000354 | isorhamnetin | GABRA1 |
| MOL000354 | isorhamnetin | MAOB |
| MOL000354 | isorhamnetin | GRIA2 |
| MOL000354 | isorhamnetin | CYP450 |
| MOL000354 | isorhamnetin | RELA |
| MOL000354 | isorhamnetin | XDH |
| MOL000354 | isorhamnetin | NCF1 |
| MOL000354 | isorhamnetin | OLR1 |
| MOL000098 | quercetin | PTGS1 |
| MOL000098 | quercetin | AR |
| MOL000098 | quercetin | PPARG |
| MOL000098 | quercetin | PTGS2 |
| MOL000098 | quercetin | HSP90 |
| MOL000098 | quercetin | PIK3CG |
| MOL000098 | quercetin | NCOA2 |
| MOL000098 | quercetin | DPP4 |
| MOL000098 | quercetin | AKR1B10 |
| MOL000098 | quercetin | PRSS1 |
| MOL000098 | quercetin | PTGS1 |
| MOL000098 | quercetin | AR |
| MOL000098 | quercetin | PPARG |
| MOL000098 | quercetin | PTGS2 |
| MOL000098 | quercetin | HSP90 |
| MOL000098 | quercetin | PIK3CG |
| MOL000098 | quercetin | NCOA2 |
| MOL000098 | quercetin | DPP4 |
| MOL000098 | quercetin | AKR1B10 |
| MOL000098 | quercetin | PRSS1 |
| MOL000098 | quercetin | RXRA |
| MOL000098 | quercetin | ACHE |
| MOL000098 | quercetin | GABRA1 |
| MOL000098 | quercetin | MAOB |
| MOL000098 | quercetin | RELA |
| MOL000098 | quercetin | EGFR |
| MOL000098 | quercetin | AKT1 |
| MOL000098 | quercetin | VEGFA |
| MOL000098 | quercetin | CCND1 |
| MOL000098 | quercetin | BCL2 |
| MOL000098 | quercetin | BCL2L1 |
| MOL000098 | quercetin | FOS |
| MOL000098 | quercetin | CDKN1A |
| MOL000098 | quercetin | EIF6 |
| MOL000098 | quercetin | BAX |
| MOL000098 | quercetin | CASP9 |
| MOL000098 | quercetin | PLAU |
| MOL000098 | quercetin | MMP2 |
| MOL000098 | quercetin | MMP9 |
| MOL000098 | quercetin | MAPK1 |
| MOL000098 | quercetin | IL10 |
| MOL000098 | quercetin | EGF |
| MOL000098 | quercetin | RB1 |
| MOL000098 | quercetin | TNF |
| MOL000098 | quercetin | JUN |
| MOL000098 | quercetin | IL6 |
| MOL000098 | quercetin | CHEK1 |
| MOL000098 | quercetin | AHSA1 |
| MOL000098 | quercetin | CASP3 |
| MOL000098 | quercetin | TP53 |
| MOL000098 | quercetin | ELK1 |
| MOL000098 | quercetin | NFKBIA |
| MOL000098 | quercetin | POR |
| MOL000098 | quercetin | ODC1 |
| MOL000098 | quercetin | XDH |
| MOL000098 | quercetin | CASP8 |
| MOL000098 | quercetin | TOP1 |
| MOL000098 | quercetin | RAF1 |
| MOL000098 | quercetin | SOD1 |
| MOL000098 | quercetin | PRKCA |
| MOL000098 | quercetin | MMP1 |
| MOL000098 | quercetin | HIF1A |
| MOL000098 | quercetin | STAT1 |
| MOL000098 | quercetin | RUNX1T1 |
| MOL000098 | quercetin | #N/A |
| MOL000098 | quercetin | CDK1 |
| MOL000098 | quercetin | HSPA5 |
| MOL000098 | quercetin | ERBB2 |
| MOL000098 | quercetin | PPARG |
| MOL000098 | quercetin | ACACA |
| MOL000098 | quercetin | HMOX1 |
| MOL000098 | quercetin | CYP3A4 |
| MOL000098 | quercetin | CYP1A2 |
| MOL000098 | quercetin | CAV1 |
| MOL000098 | quercetin | MYC |
| MOL000098 | quercetin | F3 |
| MOL000098 | quercetin | GJA1 |
| MOL000098 | quercetin | CYP1A1 |
| MOL000098 | quercetin | ICAM1 |
| MOL000098 | quercetin | IL1B |
| MOL000098 | quercetin | CCL2 |
| MOL000098 | quercetin | SELE |
| MOL000098 | quercetin | VCAM1 |
| MOL000098 | quercetin | PTGER3 |
| MOL000098 | quercetin | CXCL8 |
| MOL000098 | quercetin | PRKCB |
| MOL000098 | quercetin | BIRC5 |
| MOL000098 | quercetin | DUOX2 |
| MOL000098 | quercetin | NOS3 |
| MOL000098 | quercetin | HSPB1 |
| MOL000098 | quercetin | TGFB1 |
| MOL000098 | quercetin | SULT1E1 |
| MOL000098 | quercetin | MGAM |
| MOL000098 | quercetin | IL2 |
| MOL000098 | quercetin | NR1I2 |
| MOL000098 | quercetin | CYP1B1 |
| MOL000098 | quercetin | CCNB1 |
| MOL000098 | quercetin | PLAT |
| MOL000098 | quercetin | THBD |
| MOL000098 | quercetin | SERPINE1 |
| MOL000098 | quercetin | COL1A1 |
| MOL000098 | quercetin | IFNG |
| MOL000098 | quercetin | ALOX5 |
| MOL000098 | quercetin | PTEN |
| MOL000098 | quercetin | IL1A |
| MOL000098 | quercetin | MPO |
| MOL000098 | quercetin | TOP2A |
| MOL000098 | quercetin | NCF1 |
| MOL000098 | quercetin | ABCG1 |
| MOL000098 | quercetin | HAS2 |
| MOL000098 | quercetin | GSTP1 |
| MOL000098 | quercetin | NFE2L2 |
| MOL000098 | quercetin | NQO1 |
| MOL000098 | quercetin | TNKS |
| MOL000098 | quercetin | AHR |
| MOL000098 | quercetin | PSMD3 |
| MOL000098 | quercetin | SLC2A4 |
| MOL000098 | quercetin | COL8A1 |
| MOL000098 | quercetin | TOP2A |
| MOL000098 | quercetin | CXCL11 |
| MOL000098 | quercetin | CXCL2 |
| MOL000098 | quercetin | DCAF5 |
| MOL000098 | quercetin | NR1I3 |
| MOL000098 | quercetin | CHEK2 |
| MOL000098 | quercetin | INSR |
| MOL000098 | quercetin | CLDN4 |
| MOL000098 | quercetin | PPARA |
| MOL000098 | quercetin | PPARD |
| MOL000098 | quercetin | HSF1 |
| MOL000098 | quercetin | CRP |
| MOL000098 | quercetin | CXCL10 |
| MOL000098 | quercetin | CHUK |
| MOL000098 | quercetin | SPP1 |
| MOL000098 | quercetin | RUNX2 |
| MOL000098 | quercetin | RASSF1 |
| MOL000098 | quercetin | E2F1 |
| MOL000098 | quercetin | E2F2 |
| MOL000098 | quercetin | ACP3 |
| MOL000098 | quercetin | CTSD |
| MOL000098 | quercetin | IGFBP3 |
| MOL000098 | quercetin | IGF2 |
| MOL000098 | quercetin | CD40LG |
| MOL000098 | quercetin | IRF1 |
| MOL000098 | quercetin | ERBB3 |
| MOL000098 | quercetin | PON1 |
| MOL000098 | quercetin | DIO1 |
| MOL000098 | quercetin | PCOLCE |
| MOL000098 | quercetin | NPEPPS |
| MOL000098 | quercetin | HK2 |
| MOL000098 | quercetin | NKX3-1 |
| MOL000098 | quercetin | RASA1 |
| MOL000098 | quercetin | PRXC1A |
| MOL000098 | quercetin | GSTM1 |
| MOL000098 | quercetin | NLRP3 |
| MOL000098 | quercetin | GSTM2 |
| MOL000422 | kaempferol | NOS2 |
| MOL000422 | kaempferol | PTGS1 |
| MOL000422 | kaempferol | AR |
| MOL000422 | kaempferol | PPARG |
| MOL000422 | kaempferol | PTGS2 |
| MOL000422 | kaempferol | HSP90 |
| MOL000422 | kaempferol | PIK3CG |
| MOL000422 | kaempferol | PRKACA |
| MOL000422 | kaempferol | NCOA2 |
| MOL000422 | kaempferol | DPP4 |
| MOL000422 | kaempferol | PRSS1 |
| MOL000422 | kaempferol | PGR |
| MOL000422 | kaempferol | F2 |
| MOL000422 | kaempferol | CHRM1 |
| MOL000422 | kaempferol | NOS3 |
| MOL000422 | kaempferol | GABRA2 |
| MOL000422 | kaempferol | ACHE |
| MOL000422 | kaempferol | SLC6A2 |
| MOL000422 | kaempferol | CHRM2 |
| MOL000422 | kaempferol | ADRA1B |
| MOL000422 | kaempferol | GABRA1 |
| MOL000422 | kaempferol | TOP2A |
| MOL000422 | kaempferol | F7 |
| MOL000422 | kaempferol | CAMKK2 |
| MOL000422 | kaempferol | RELA |
| MOL000422 | kaempferol | IKBKB |
| MOL000422 | kaempferol | AKT1 |
| MOL000422 | kaempferol | BCL2 |
| MOL000422 | kaempferol | BAX |
| MOL000422 | kaempferol | TNF |
| MOL000422 | kaempferol | JUN |
| MOL000422 | kaempferol | AHSA1 |
| MOL000422 | kaempferol | CASP3 |
| MOL000422 | kaempferol | MAPK8 |
| MOL000422 | kaempferol | XDH |
| MOL000422 | kaempferol | MMP1 |
| MOL000422 | kaempferol | STAT1 |
| MOL000422 | kaempferol | CDK1 |
| MOL000422 | kaempferol | PPARG |
| MOL000422 | kaempferol | HMOX1 |
| MOL000422 | kaempferol | CYP3A4 |
| MOL000422 | kaempferol | CYP1A2 |
| MOL000422 | kaempferol | CYP1A1 |
| MOL000422 | kaempferol | ICAM1 |
| MOL000422 | kaempferol | SELE |
| MOL000422 | kaempferol | VCAM1 |
| MOL000422 | kaempferol | NR1I2 |
| MOL000422 | kaempferol | CYP1B1 |
| MOL000422 | kaempferol | ALOX5 |
| MOL000422 | kaempferol | HAS2 |
| MOL000422 | kaempferol | GSTP1 |
| MOL000422 | kaempferol | AHR |
| MOL000422 | kaempferol | PSMD3 |
| MOL000422 | kaempferol | SLC2A4 |
| MOL000422 | kaempferol | NR1I3 |
| MOL000422 | kaempferol | INSR |
| MOL000422 | kaempferol | DIO1 |
| MOL000422 | kaempferol | PPP3CA |
| MOL000422 | kaempferol | PRXC1A |
| MOL000422 | kaempferol | GSTM1 |
| MOL000422 | kaempferol | GSTM2 |
| MOL000422 | kaempferol | AKR1C3 |
| MOL000422 | kaempferol | SLPI |
| MOL002322 | isovitexin | PTGS2 |
| MOL002322 | isovitexin | AR |
| MOL002322 | isovitexin | TOP2A |
| MOL002322 | isovitexin | RELA |
| MOL002322 | isovitexin | IKBKB |
| MOL002322 | isovitexin | TNF |

**NLRP3 knockdown efficiency verification**

The knockdown efficiency of NLRP3 was verified by qRT-PCR and WB methods. The results proved that the knockdown rate of the NLRP3 gene reached 79.1% and that of the NLRP3 protein reached 78.2%. The results fully demonstrate the success of NLRP3 gene knockdown.


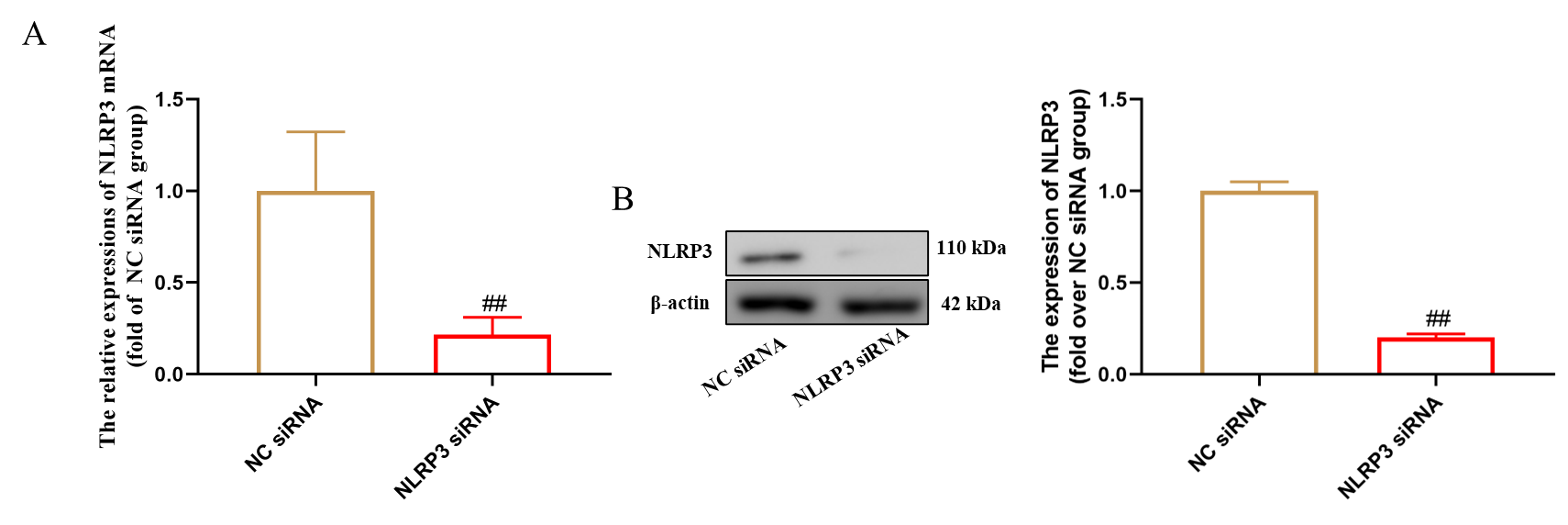


**Supplementary Figure.1** NLRP3 knockdown efficiency verification. (A) qRT-PCR and (B) WB were used to detected the levels of *NLRP3* mRNAs after NLRP3 knock-down.

**Molecular docking results**

The binding energies of Astragalin, Kaempferol 3-O-glucoside-7-O-rhamnoside and Kaempferol 3-O-rutinoside to NLRP3 were 0.38, 5.96 and -3.45, respectively. The molecular docking results of the above-mentioned compounds with NLRP3 show a relatively weak binding ability.


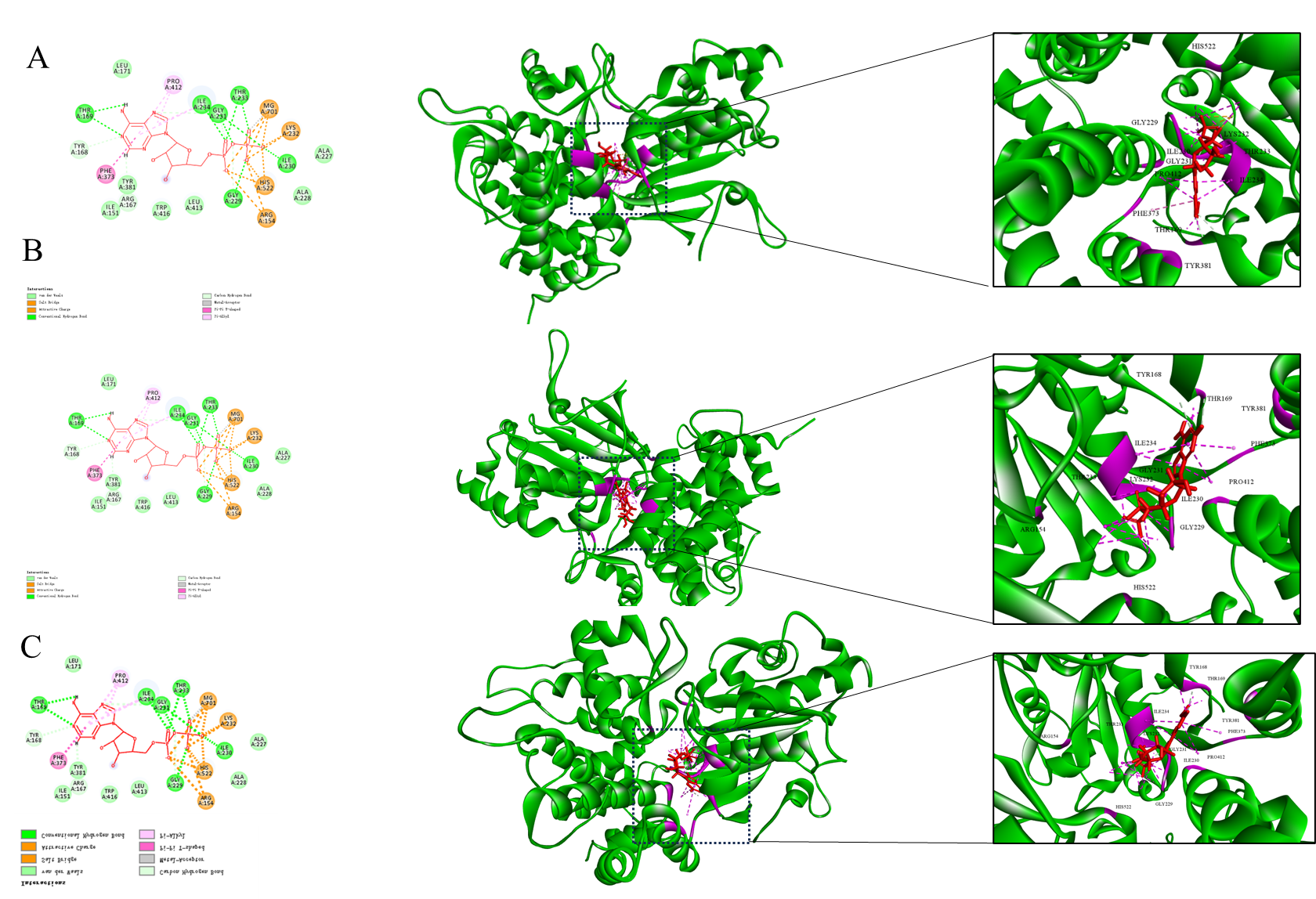


**Supplementary Figure.2** The molecular docking images of NLRP3 and Kaempferol 3-O-glucoside-7-O-rhamnoside, Kaempferol-3-O-rutinoside and Astragalin. 2D and 3D images and zoom in on specific areas of NLRP3 with (A) Astragalin, (B) Kaempferol 3-O-glucoside-7-O-rhamnoside and (C) Kaempferol-3-O-rutinoside.
